# Precise Control of Molecular Weight Characteristics of Charge‐Shifting Poly(2‐(*N*,*N*‐Dimethylamino)Ethylacrylate) Synthesized by Reversible Addition‐Fragmentation Chain Transfer Polymerization

**DOI:** 10.1002/marc.202400640

**Published:** 2024-11-03

**Authors:** Radoslava Sivkova, Rafal Konefal, Libor Kostka, Richard Laga, Gabriela S. García‐Briones, Olga Kočková, Ognen Pop‐Georgievski, Dana Kubies

**Affiliations:** ^1^ Department of Chemistry and Physics of Surfaces and Biointerfaces Institute of Macromolecular Chemistry Czech Academy of Sciences Heyrovsky sq. 2, Prague 6 Prague 162 06 Czech Republic; ^2^ Department of Structural Analysis Institute of Macromolecular Chemistry Czech Academy of Sciences Heyrovsky sq. 2, Prague 6 Prague 162 06 Czech Republic; ^3^ Department of Biomedical Polymers Institute of Macromolecular Chemistry Czech Academy of Sciences Heyrovsky sq. 2, Prague 6 Prague 162 06 Czech Republic; ^4^ Department of Polymer and Colloid Immunotherapeutics Institute of Macromolecular Chemistry Czech Academy of Sciences Heyrovsky sq. 2, Prague 6 Prague 162 06 Czech Republic; ^5^ Analytical Chemistry Laboratory Institute of Macromolecular Chemistry Czech Academy of Sciences Heyrovsky sq. 2, Prague 6 Prague 162 06 Czech Republic

**Keywords:** charge‐shifting polymer, DMAEA, hydrolysis, polycation, RAFT polymerization

## Abstract

Poly(2‐(*N*,*N*‐dimethylamino)ethyl acrylate) (PDMAEA) is a promising charge‐shifting polycation with the capacity to form a range of morphologically distinct polyelectrolyte assemblies. Nevertheless, the basic character of the monomer and its hydrolytic instability impedes its controlled synthesis to higher molecular weight (MW). Herein, the reversible addition‐fragmentation chain transfer polymerization of DMAEA is reported using a tert‐butanol/V70 initiator/trithiocarbonate‐based chain transfer agent (CTA) polymerization setup. The CTA instability is demonstrated in the presence of the unprotonated tertiary amino group of the DMAEA monomer, which limits the control over the conversion and MW of the polymer. In contrast, the shielding of the amino groups by their protonation leads to polymerization with high conversions and excellent control over MWs of polymer up to 100 000 g mol^−1^. Hydrolytic degradation study at pH values ranging from 5 to 9 reveals that both basic and protonated PDMAEA undergo a pH‐dependent hydrolysis. The proposed polymerization conditions provide a means of synthesizing PDMAEA with well‐controlled characteristics, which are beneficial for controlling the complexation processes during the formation of various polyelectrolyte assemblies.

## Graphical abstract

Control of the RAFT polymerization process of 2‐(*N,N*‐dimethylamino)ethyl acrylate (DMAEA) is limited by the decomposition of trithiocarbonate end groups of growing polymer chains caused by amino groups of DMAEA, whereas shielding of amino groups by their protonation leads to the polymers with well‐controlled molecular weights up to 100 kDa. Both basic and protonated PDMAEA, known as charge‐shifting polymers, undergo pH‐dependent hydrolysis.

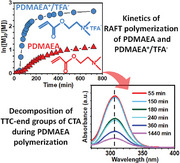

## 1 Introduction

2‐(*N*,*N*‐dimethylamino)ethyl acrylate (DMAEA) and 2‐(*N*,*N*‐dimethylamino)ethyl methacrylate (DMAEMA) are well‐known monomers containing tertiary amino group that can be converted into quaternary ammonium salts by reaction with alkyl halides, or protonated with strong organic and inorganic acids.^[^ ^1^, ^2^ ^]^ This ability makes them promising molecules for the preparation of polymers that are commonly used as carriers of negatively charged nucleic acids in gene delivery.^[^ ^3^, ^4^ ^]^ DMAEMA is a stable, reactive methacrylic monomer and its polymerization in both free radical and controlled radical methods has been well studied over the years.^[^ ^5^, ^6^ ^]^ In contrast, DMAEA is significantly less stable, and both the monomer and its polymers are known to undergo self‐catalyzed hydrolysis of ester bonds in the side chains to form acrylic acid monomer units and *N*,*N*‐dimethyl‐aminoethanol.^[^ ^7^, ^8^, ^9^, ^10^, ^11^ ^]^ The ability to cleave side groups categorizes PDMAEA as a significant member of the class of charge‐shifting polycations that can convert their charges in response to external stimulus.^[^ ^4^, ^12^ ^]^ Charge‐shifting polycations have attracted increasing interest in gene delivery over the past decade. The controlled hydrolysis of the cationic side groups of PDMAEA may serve as a means of releasing negatively charged nucleic acids that are electrostatically complex with PDMAEA.^[^ ^4^, ^13^ ^]^ Nevertheless, the hydrolytic instability of both the monomer and polymer significantly complicates the polymerization process.

DMAEA‐based polymers are most commonly synthesized by free‐radical polymerization methods in which the MWs and dispersity (*Ð*) of the resulting polymers are not well‐controlled.^[^ ^8^, ^14^ ^]^ In order to achieve better control over the structural parameters of the polymers, controlled polymerization processes, such as reversible addition–fragmentation chain transfer polymerization (RAFT) or atom‐transfer radical polymerization (ATRP), must be applied. However, DMAEA is not a good candidate for ATRP due to the possible side reactions between the tertiary amino group of the monomer and the end halide group in the growing polymer chains.^[^ ^9^, ^15^ ^]^ In the literature, most studies report on RAFT polymerizations of DMAEA with the products of MW up to 8000 g mol^−1^ to form polyplexes with siRNA or DNA.^[^ ^4^, ^9^, ^10^, ^13^, ^16^ ^]^ There is a limited number of papers on the RAFT polymerization of PDMAEA with MW up to 15 000 g mol^−1^.^[^ ^11^, ^17^, ^18^ ^]^ The RAFT polymerization of DMAEA in basic form was carried out using mainly trithiocarbonate (TTC) CTAs and 2,2′‐azobis(isobutyronitrile) as an initiator in organic solvents such as dioxane, DMF, 2‐butanone, or toluene. The polymerizations resulted in homopolymers^[^ ^2^, ^3^, ^16^, ^17^, ^19^ ^]^ and statistical and block copolymers^[^ ^2^, ^18^, ^19^, ^20^, ^21^, ^22^ ^]^ with *Ð* of ≈1.3, and the conversions ranging from 20% to 75%. Recently, a light‐induced RAFT polymerization of DMAEA resulted in conversions between 50% and 60% and MWs between 7000 and 20 000 g mol^−1^.^[^ ^23^ ^]^

The RAFT polymerization of monomers containing nucleophilic primary or secondary amine substituents is a challenging process due to their reactivity toward sulfur‐containing groups of the CTA.^[^ ^24^, ^25^ ^]^ The polymerization of such monomers is typically carried after the protection of the amino groups with *tert*‐butoxycarbonyl groups,^[^ ^26^, ^27^ ^]^ or, in the case of primary amines, the monomers can be used in their hydrochloride form.^[^ ^28^, ^29^ ^]^ Furthermore, maintaining a pH of ≈5 and reducing the reaction temperature also minimizes the possibility of aminolysis of the CTA.^[^ ^30^ ^]^ However, such complications have been rarely reported for monomers containing tertiary amino groups.^[^ ^31^ ^]^

Nevertheless, some difficulties associated with the synthesis of PDMAEA using RAFT polymerization were pointed out by Li et al., who observed a gradual decomposition of TTC‐based CTAs in the presence of the tertiary amino group of DMAEA.^[^ ^19^ ^]^ The decomposition of the monomer and CTA was also reported for 2‐(*N*,*N*‐diethylamino)ethyl acrylate (DEAEA) RAFT polymerization in the presence of 4‐cyano‐4‐(phenylcarbonothioylthio) pentanoic acid (CTP) as CTA in a water/ethanol mixture.^[^ ^32^ ^]^ The strategy proposed to overcome the DEAEA hydrolysis in aqueous conditions and, at the same time, the CTP degradation caused by the presence of monomer with basic amino groups was the protonation of DEAEA with trifluoracetic acid (TFA) prior to polymerization. This approach resulted in an increase in conversion from 10% to almost 80%, but MWs higher than 30 000 g mol^−1^ were not achieved. DMAEA was also polymerized in the presence of CTP in a water/dioxan solvent system at a pH of 3–4, adjusted with hydrochloric acid, to prevent the degradation of the hydrolytically labile ester groups of DMAEA.^[^ ^8^, ^33^ ^]^ These conditions led to conversions close to 90% and the polymers of MW approaching 30 000 g mol^−1^.

In addition to its use in nucleic acid delivery, PDMAEA has the potential to form ultrathin polyelectrolyte multilayers with negatively charged polysaccharides, peptides, or proteins, which may find applications in the construction of biomaterial coatings or biosensors. In this case, the higher MWs of the polymer components are preferable due to the increased stability of such polyelectrolyte films.^[^ ^34^, ^35^, ^36^ ^]^ Until now, the published works on RAFT polymerization did not report MWs of PDMAEA approaching 100 000 g mol^−1^. Furthermore, only a few attempts of DMAEA homopolymerization have been performed, yielding polymers with MW below 10 000 g mol^−1^. Additionally, the polymerization of the basic (DMAEA) and protonated (DMAEA^+^/TFA^−^) monomer in non‐aqueous conditions, which avoids the monomer hydrolysis, has never been compared using the same polymerization system.

**Figure 1:**
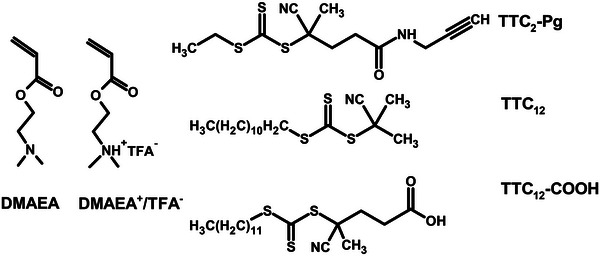
Structure of DMAEA and DMAEA^+^/TFA^−^ monomers and chain transfer agents 2‐cyano‐5‐oxo‐5‐[(prop‐2‐yn‐1‐yl)amino]pentan‐2‐yl ethyl carbonotrithioate (TTC_2_‐Pg), 2‐cyano‐2‐propyl‐dodecyl trithiocarbonate (TTC_12_) and 4‐cyano‐4‐[(dodecylsulfanylthiocarbonyl)sulfanyl]pentanoic acid (TTC_12_‐COOH).

In this contribution, we describe a solvent/initiator/CTA RAFT system designed to enable the polymerization of both the basic and protonated forms of DMAEA. To study the effect of CTA structure on kinetics, three TTC‐based CTAs, i.e., 2‐cyano‐2‐propyl‐dodecyl trithiocarbonate (TTC_12_), 4‐cyano‐4‐[(dodecylsulfanylthiocarbonyl)sulfanyl]pentanoic acid (TTC_12_‐COOH) and 2‐cyano‐5‐oxo‐5‐[(prop‐2‐yn‐1‐yl)amino]pentan‐2‐yl ethyl carbonotrithioate (TTC_2_‐Pg, see the Supporting Information for the synthesis) were used. The structures of the monomers and CTAs are depicted in **Figure** **1**. We studied the controlled character of the polymerization of both monomers by aiming at products with different MW by varying the monomer/CTA ratio in the polymerization feed. Ultimately, to evaluate the effect of protonation on the hydrolytic stability of the polymer side chains, a hydrolysis study was carried out at various pH values for both polycations.

## 2 Results and Discussion

Based on our previous experience with RAFT polymerization of (meth)acrylamides by TTC‐based CTAs,^[^ ^27^, ^37^ ^]^ we have employed a mixture of *tert*‐butanol with 10 (for DMAEA) and 20 (for DMAEA^+^/TFA^−^) vol.% of dimethylacetamide (DMA) as the solvent system to ensure the CTA, initiator and product solubility in the polymerization mixture. The oil‐soluble azo initiator 2,2′‐azobis(4‐methoxy‐2,4‐dimethylvaleronitrile) (V70) allowed polymerization to be performed at 40 °C, which is advantageous in terms of limiting side reactions during the RAFT process.^[^ ^38^ ^]^ For the experiments of the MW dependence on the monomer/CTA ratio, we used TTC_2_‐Pg as the CTA because end propargyl groups can be used for subsequent post‐polymerization modification via Huisgen type “click” chemistry. In kinetic studies, a model [M]/[CTA]/[I] molar ratio of 260/1/0.2 was used to obtain an MW approaching 40 000 g mol^−1^ for the basic DMAEA monomer.

The polymerization process was investigated through two approaches: a) in situ monitoring of the polymerization in a *tert*‐butanol‐*d*_10_/DMA mixture by ^1^H NMR analysis (Figures S1 and S2, Supporting Information) and b) analysis of aliquots taken from the polymerization mixture with increasing polymerization time. The evolution of monomer conversion, as determined by ^1^H NMR analysis in acetone‐*d*_6_ (Figures S3 and S4, Supporting Information), was correlated with MWs and *Ð* of the products monitored by SEC‐MALS analysis. Details about the experimental setup, synthesis, and analytical methods employed are presented in the Supporting Information.

### 2.1 RAFT Polymerization of Basic and Protonated DMAEA

**Figure 2:**
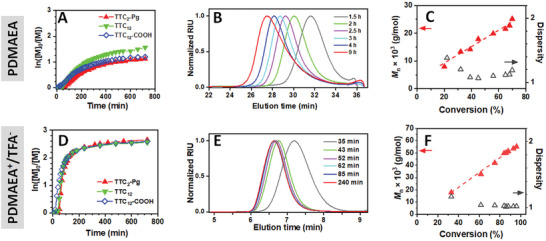
Characteristics of A–C) PDMAEA and D–F) DMAEA^+^/TFA^−^ polymerizations: Kinetic plots of ln([M_0_]/[M]) versus time (A, D) (in situ ^1^H NMR analysis in *tert*‐butanol‐*d*_10_); SEC chromatograms of the polymerization mixtures with increasing polymerization time, CTA: TTC_2_‐Pg, (B, E); Dependence of *M*_n_ (red filled triangle) and dispersity (empty black triangle) on the conversion, CTA: TTC_2_‐Pg, (C, F). All polymerizations performed for [M]/[CTA] = 260, [CTA]/[V70] = 5, [M] = 2 m in *tert*‐butanol/DMA 10 vol.% (DMAEA) and *tert*‐butanol/DMA 20 vol.% (DMAEA^+^/TFA^−^) solvent mixtures, 40 °C. Details about both SEC analyses are presented in the Supporting Information.

In the case of the basic DMAEA monomer, the kinetic plots of ln([M_0_]/[M]) versus time showed that the course of all three polymerizations was comparable (**Figure** **2**A), although minor differences were observed. For example, depending on the CTA used, the induction period ranged from 20 min to 1 h, with the shortest being for TTC_12_‐COOH. The length of the induction period can be attributed to the stability of the respective CTA radicals or the efficiency of re‐initiation. After the induction period, the kinetics exhibited a pseudo‐first‐order character until 200 min (≈50% conversion) in all the cases (Figure S5, Supporting Information). Afterward, the polymerization slowed, and the conversion reached a plateau. After 12 h, the highest conversion of 70% was achieved for TTC_12_, followed by 65% for TTC_12_‐COOH, and 60% for TTC_2_‐Pg. The polymer chains grew continuously with proceeding polymerization (Figure 2B), with a slight tailing at higher polymerization times, but with an unimodal distribution of MWs. Indeed, the evolution of the MW with conversion (Figure 2C) was linear until 65% conversion, and *Ð* of the products did not exceed the value of 1.3, typically reported for controlled RAFT polymerization.

In the case of the protonated DMAEA^+^/TFA^−^ monomer, the kinetic plots of ln([M_0_]/[M]) versus time showed almost identical polymerization courses for all three CTAs studied (Figure 2D). A shorter induction period of 20 min was followed by a rapid increase in conversion, with pseudo‐first‐order kinetics until 80 min of polymerization (Figure S5, Supporting Information), indicating that the concentration of radical species was constant until ≈80% conversion. In contrast to the DMAEA polymerization, the conversion reached more than 90% within 3 h. Again, the growing polymer chains had an unimodal distribution of MWs (Figure 2E). The observed linear growth of the MW with increasing monomer conversion and the very low *Ð* of the polymers below 1.1 (Figure 2F), together with the high conversions achieved, indicate well‐controlled RAFT polymerizations of DMAEA^+^/TFA^−^. For both monomers, the determined MW values were in good agreement with the theoretical values (27 200 g mol^−1^ for DMAEA, and 66 900 g mol^−1^ for DMAEA^+^/TFA^−^) and the conversions achieved (Table S1, Supporting Information, entries for the [M]/[TTC_2_‐Pg] ratio of 260).

**Figure 3:**
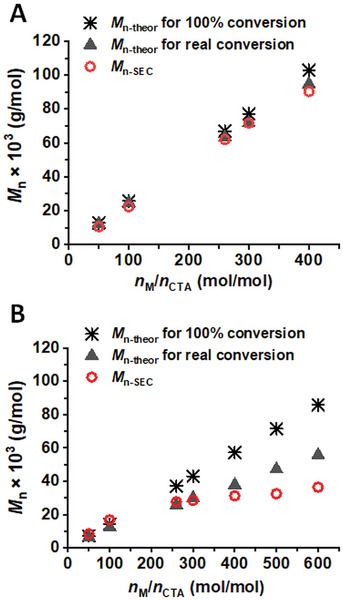
Dependence of *M*_n_ on a [M]/[TTC_2_‐Pg] ratio for A) the protonated PDMAEA^+^/TFA^−^ and B) basic PDMAEA polymers: theoretical *M*_n_ calculated for 100% conversion (black asterisk); theoretical *M*_n_ calculated for a real conversion determined by ^1^H NMR analysis in acetone‐*d*_6_ (black filled triangle), and *M*_n_ determined by SEC analysis (red open circle). [TTC_2_‐Pg]/[V70] = 5.

Furthermore, the [M]/[CTA] ratio was varied from 50 to 600 in order to obtain the products with MW ranging from 10 000 to 100 000 g mol^−1^ (Table S1, Supporting Information). In the case of DMAEA^+^/TFA^−^, all the products exhibited a narrow unimodal distribution of MWs (Figure S6A, Supporting Information), with conversions of ≈90% and the determined MWs that corresponded to the theoretical values considering the respective conversions, and with *Ð* below 1.2 even for high MWs (**Figure** **3**A; Table S1, Supporting Information). Such good control over the polymer parameters indicates excellent control over the polymerization process of DMAEA^+^/TFA^−^.

In contrast, for PDMAEA, the conversion decreased from 89% to 65% with an increasing [M]/[CTA] ratio, and the control over MW was lost for a [M]/[CTA] ratio greater than 200 (Figure 3B; Table S1, Supporting Information). All products exhibited an unimodal distribution of MWs (Figure S6B, Supporting Information), albeit with a slight increase in *Ð* with an increasing [M]/[CTA] ratio, which still did not exceed 1.3. An additional variation of the polymerization conditions, specifically the [CTA]/[I] ratio and a decrease in the monomer concentration from 2 to 0.7 mol L^−1^ did not affect the discrepancies observed between the theoretical and determined MW, nor did they lead to an increase in the conversions (Figure S7, Supporting Information). The results obtained led us to conclude that the differences in the polymerization course between the protonated and basic forms of DMAEA can be attributed to the stability of CTA in the presence of the monomer. It has been demonstrated that the tertiary amino groups can dramatically affect the stability of the TTC‐based CTAs, leading to their gradual decomposition.^[^ ^19^ ^]^ In contrast to DMAEA with unprotected reactive amino groups, the protonation of the tertiary amine with TFA has the potential to prevent side reactions and maintain control over the DMAEA polymerization.

**Figure 4:**
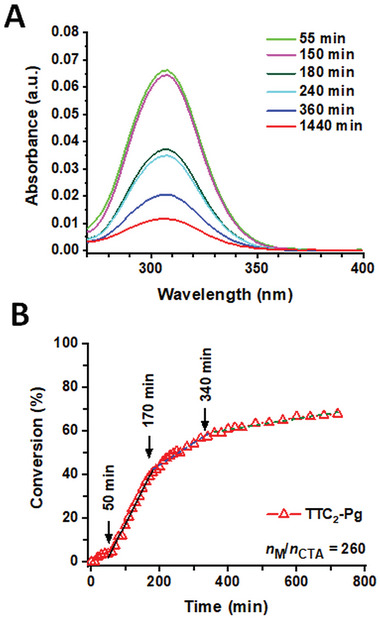
Polymerization of basic DMAEA: A) Absorption band intensity at a wavelength of 307 nm (corresponding to the polymer TTC‐containing CTA end groups) at various polymerization times; B) Dependence of conversion on polymerization time (lines–fittings). Polymerization conditions: in *tert*‐butanol/DMA 10 vol.%, 40 °C, 12 h, [DMAEA] = 2 m; [TTC_2_‐Pg]/[V70] = 5.

To monitor the stability of TTC_2_‐Pg during the polymerization of the basic DMAEA, we analyzed the UV–vis spectra corresponding to the maximum of the polymer peak in SEC chromatograms, obtained from the diode array detector of the SEC instrumental set‐up. In particular, we focused on the band with an absorption maximum at a wavelength of 307 nm, which arises from the TTC moiety at the end of the polymer chain. We followed the changes in absorbance with proceeding polymerization and compared them with the evolution of conversion (**Figure** **4**). The absorbance decreased with increasing polymerization time, and the decrease had a stepwise character (Figure 4A). Specifically, the absorbance started to decrease very slowly after ≈50 min, followed by the most intensive decrease after 150 min, with relative stability between 180 and 240 min, and the final intensive decrease after 360 min of polymerization. Importantly, comparable turning points were observed in the plot of conversion versus polymerization time (Figure 4B); the plot can be divided into three regions fitted with the pseudo‐first‐order kinetic, the first starting at 50 min, then gradually slowing after 170 min, and a final region starting from 340 min onward, indicating a very slow polymerization. This empirical observation suggests that the polymerization rate is strongly influenced by the decomposition of TTC endgroups. Our findings correlate with the previously reported decomposition of TTC‐based CTAs during the DMAEA polymerization in DMF, followed by online monitoring of the end groups of the growing polymer chains.^[^ ^19^ ^]^ In contrast, during the polymerization of the protonated DMAEA^+^/TFA^−^ monomer, no obvious relation of the intensity of the UV–vis band at 307 nm with increasing polymerization time was observed, indicating the stability of the TTC active ends of the growing polymer chains (Figure S8, Supporting Information).

The monomer reactivity may also be an important factor contributing to the polymerization rate. As expected, the course of free radical polymerization did not markedly differ for both monomers (Figure S9A, Supporting Information). The results obtained suggest that the present TFA^−^ counterions only slightly enhance the propagation rate likely by screening the repulsive electrostatic interactions between the positively charged species in the monomers, the growing radical chains, and the polymer chains.

In addition to the demonstrated CTA degradation, the causes of the observed kinetics of the DMAEA RAFT polymerization, which lie in the polymerization mechanism, must also be considered. Figure S9B (Supporting Information) depicts the monomer conversion as a function of polymerization time for the free radical and RAFT polymerization of DMAEA using the same experimental conditions. A comparison of the course of both polymerizations during the first 150 min, when the degradation of CTA is not expected to be significant (Figure 4), revealed that the free radical polymerization is considerably faster, reaching 73% conversion compared to 33% determined for the RAFT polymerization. This observation corresponds to the typical characteristics of RAFT polymerization. The RAFT polymerization mechanism represents a very complex process of individual polymerization stages.^[^ ^31^, ^38^ ^]^ The observed rate‐retarded RAFT polymerization of DMAEA can result from several factors.^[^ ^39^, ^40^ ^]^ For example, RAFT adduct radicals formed during the pre‐equilibrium are sufficiently thermodynamically favorable, which leads to their too slow fragmentation and thus to their low concentration in the polymerization system.^[^ ^41^, ^42^, ^43^ ^]^ In addition, a low affinity of leaving radical R⋅ groups from CTA (i.e., from a S = C(Z)S‐R structure) toward the monomer results in a slow re‐initiation step, and thus an insufficient amount of monomer radicals available to propagate (i.e., degradative chain transfer).^[^ ^39^, ^44^ ^]^ The termination can involve intermediated radical termination (ITR) processes, whereby all radicals present in the system could terminate with RAFT intermediate (adduct) radicals. Consequently, ITR is also considered one of the reasons for rate‐retarded polymerization.^[^ ^38^, ^39^ ^]^

### 2.2 Hydrolysis Study

**Figure 5:**
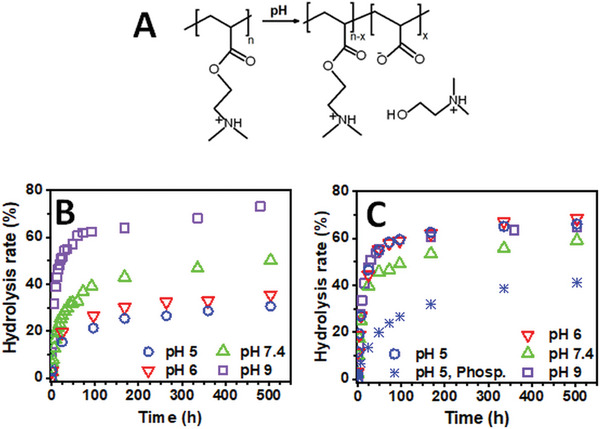
A) Scheme of the PDMAEA^+^/TFA^−^ hydrolysis, and hydrolysis of B) 0.5% wt. PDMAEA^+^/TFA^−^ and C) PDMAEA solutions in 100 mm acetate (pH 5, blue empty circle; pH 6, red empty triangle), phosphate (pH 7.4, green empty triangle) and borate‐buffered (pH 9, purple empty square) D_2_O at 37 °C. For PDMAEA, phosphate (blue asterisk) and citrate/phosphate (Figure S11, Supporting Information) buffers of pH 5 were also used. (^1^H NMR analysis).

The charge‐shifting behavior of PDMAEA, which results from the hydrolysis of the ester group of the monomer (**Figure** **5**A), provides a means of controlling the disruption of polyelectrolyte complexes of PDMAEA with polyanions^[^ ^7^, ^16^, ^33^ ^]^ and of lowering the charge density, which may result in a reduction in polycation toxicity. For comparison purposes, the hydrolysis study was carried out at pH values ranging from 5 to 9, using the buffers previously described in the literature^[^ ^7^, ^8^ ^]^ (see Figure 5 for the buffers used). On the other hand, in contrast to the polymer concentration of 1 to 4 mg mL^−1^ used in the previously reported hydrolysis studies,^[^ ^2^, ^7^, ^8^, ^10^, ^18^, ^46^ ^]^ we selected a concentration of 0.5 mg mL^−1^ to ensure sufficient buffer capacity of the buffers used, as also recently suggested by Ross et al.^[^ ^8^, ^45^ ^]^

The rate of hydrolysis of the protonated PDMAEA^+^/TFA^−^ performed at 37 °C increased with increasing pH (Figure 5B), reflecting the hydroxide‐mediated mechanism of ester hydrolysis. (Figure S10 Supporting Information shows representative ^1^H NMR spectra for the PDMAEA^+^/TFA^−^ hydrolysis at pH 7.4.) A similar trend was recently observed for the hydrolysis of PDMAEA protonated with hydrochloric acid at 22 °C and its copolymers with *N*‐(3‐aminopropyl)methacrylamide hydrochloride at 37 °C.^[^ ^8^, ^45^ ^]^

In contrast, the basic PDMAEA has been repeatedly reported to undergo self‐catalyzed hydrolysis independent of pH.^[^ ^7^, ^9^, ^10^, ^46^ ^]^ Although we also initially determined comparable hydrolysis profiles at pH 5, 6, and 9 (Figure 5C), a slightly different hydrolysis pattern was observed at pH 7.4, suggesting that the acetate buffers of pH 5 and 6 may have insufficient buffering capacity at higher pH values. Indeed, the pH of the PDMAEA solutions after three weeks of incubation in both 0.1 m acetate buffers was ≈9.3, and the buffering capacity did not increase even when the acetate buffer (pH 5) of tenfold concentration was used (Table S2, Figure S11, Supporting Information). This observation suggests that PDMAEA (with a p*K*_a_ of DMAEA of ≈8.3^[^ ^47^ ^]^) can itself act as a buffer due to the presence of tertiary amines in its structure. On the other hand, when the study was conducted at pH 5 using phosphate and citrate‐phosphate buffers, the hydrolysis was found to be significantly slower than in the acetate buffer at pH 5 and 6, as well as in the buffers at pH 7.4 and 9 (Figure 5C; FigureS11, Supporting Information). Our findings thus demonstrate that the hydrolysis of basic PDMAEA is also pH‐dependent when selecting appropriate experimental conditions.

## 3 Conclusion

In summary, the studied polymerization system allows for the RAFT polymerization of DMAEA in polar non‐aqueous conditions. The degradation of TTC groups of CTA, caused by the tertiary amino groups of the basic DMAEA, led to lower conversions and a loss of control over the MWs of PDMAEA above 20 000 g mol^−1^. Conversely, the controlled protonation of the amino groups of DMAEA with a counterion, in this case, TFA, prior to polymerization prevented the amine‐triggered decomposition of the CTA. This resulted in a prolonged, controlled RAFT process of up to 90% conversions, with excellent control over predetermined molecular weights (here from 10 000 to 100 000 g moL^−1^) and with narrow *Ð*. In addition, the comparative hydrolysis study revealed that not only the protonated PDMAEA^+^/TFA^−^ but also the basic PDMAEA undergoes pH‐dependent hydrolysis when non‐acetate buffers are employed. This finding differs from the conclusions of previously published studies. The proposed RAFT polymerization conditions provide a means of synthesizing PDMAEA with well‐controlled characteristics, which are beneficial for controlling the complexation process during the formation of polyelectrolyte complexes.

## Conflict of Interest

The authors declare no conflict of interest.

## Author Contributions

The manuscript was equally written by R. Sivkova and D. Kubies. All authors have approved the final version of the manuscript.

## Supporting information



Supporting Information

## Data Availability

The data that support the findings of this study are available from the corresponding author upon reasonable request.
