# Supplementary material for: Precise Control of Molecular Weight Characteristics of Charge‐Shifting Poly(2‐(N,N‐Dimethylamino)Ethylacrylate) Synthesized by Reversible Addition‐Fragmentation Chain Transfer Polymerization
Source: Macromol Rapid Commun. 2024 Nov 3;46(2):2400640. doi: 10.1002/marc.202400640 (PMC11756865; doi:10.1002/marc.202400640)
Supplement: Supplementary file 1 — Supporting Information [file MARC-46-2400640-s001.docx]

Precise control of molecular weight characteristics of Charge-Shifting poly(2-(*N,N*-dimethylamino)ethyl acrylate) synthesized by reversible addition-fragmentation chain-transfer polymerization

RADOSLAVA SIVKOVA ^a^, RAFAL KONEFAL ^a^, LIBOR KOSTKA ^a^, RICHARD LAGA ^a^, GABRIELA S. GARCÍA-BRIONES ^a^, OLGA KOČKOVÁ ^a^, OGNEN POP-GEORGIEVSKI ^a^, DANA KUBIES ^a,^*

Institute of Macromolecular Chemistry, v.v.i., Czech Academy of Sciences, Heyrovsky sq. 2, 162 06 Prague 6, Czech Republic

* Corresponding author: Dana Kubies, e-mail: [kubies@imc.cas.cz](mailto:kubies@imc.cas.cz)

Content

[**EXPERIMENTAL PART** 3](#_Toc180747940)

[1. Materials 3](#_Toc180747941)

[2. Synthesis of 2-Cyano-5-oxo-5-[(prop-2-yn-1-yl)amino]pentan-2-yl ethyl carbonotrithioate (TTC_2_-Pg) 3](#_Toc180747942)

[3. Reversible addition–fragmentation chain transfer (RAFT) polymerization. 4](#_Toc180747943)

[3.1. DMAEA monomer 4](#_Toc180747944)

[3.2. DMAEA^+^/TFA^-^ monomer 4](#_Toc180747945)

[4. *In situ* monitoring of RAFT polymerization by ^1^H-NMR 5](#_Toc180747946)

[5. Determination of monomer conversion by ^1^H-NMR analysis in acetone-*d_6_* 5](#_Toc180747947)

[6. Size exclusion chromatography analysis 6](#_Toc180747948)

[7. Hydrolysis studies 6](#_Toc180747949)

[**RESULTS** 8](#_Toc180747950)

[**Fig. S1** ^1^H-NMR spectra of the PDMAEA polymerization solution in *tert-*butanol-*d*_10_ 8](#_Toc180747951)

[**Fig. S2** ^1^H-NMR spectra of the PDMAEA^+^/TFA^-^ polymerization solution in a *tert-*butanol-*d*_10_/TFA-*d*_1_ mixture 9](#_Toc180747952)

[**Fig. S3** ^1^H-NMR spectra of the PDMAEA polymerization solution in acetone-*d*_6_ 10](#_Toc180747953)

[**Fig. S4** ^1^H-NMR spectra of the PDMAEA^+^/TFA^-^ polymerization solution in acetone-*d*_6_ 10](#_Toc180747954)

[**Fig. S5** The linear fitting of the kinetic plots of ln([M_0_]/[M]) versus time for PDMAEA (A-C) and PDMAEA^+^/TFA^-^ (D-F) 11](#_Toc180747955)

[**Table S1** Effect of [M]/[TTC_2_-Pg] molar ratio on the molecular weight of PDMAEA and PDMAEA^-^/TFA^+^. 12](#_Toc180747956)

[**Fig. S6** Normalized SEC chromatograms of protonated PDMAEA^+^/TFA^-^ (A) and unprotonated PDMAEA (B) polymers prepared at a [M]/[TTC_2_-Pg] ratio 13](#_Toc180747957)

[**Fig. S7** Polymerization of the unprotonated PDMAEA monomer. Dependence of monomer conversion () and *M*_n_ () of products on the [TTC_2_-Pg]/[V70] ratio (A) and the DMAEA concentration in polymerization mixture (B) 13](#_Toc180747958)

[**Fig. S8** Polymerization of the protonated DMAEA^+^/TFA^-^. Absorption band intensity at a wavelength of 307 nm (corresponding to the polymer TTC-containing CTA end groups) at various polymerization times. 14](#_Toc180747959)

[**Fig. S9** Free radical polymerization of DMAEA and DMAEA^+^/TFA^-^ monomers in *tert*-butanol/DMA with V70 initiator: 14](#_Toc180747960)

[**Fig. S10** ^1^H-NMR spectra of PDMAEA^+^/TFA^-^ in a phosphate-buffered D_2_O solution (pH 7.4) 15](#_Toc180747961)

[**Table S2** The pH values of PDMAEA and PDMAEA^+^/TFA^-^ solutions in 0.1 M buffers of various pH at 37 °C after 3-week hydrolytic degradation. 16](#_Toc180747962)

[**Fig. S11** Hydrolysis of a 0.5% wt. PDMAEA solution at pH 5 using 0.1 M acetate, phosphate and citrate-phosphate-buffered D_2_O, and 1 M acetate-buffered D_2_O at 37 °C. 16](#_Toc180747963)

# **EXPERIMENTAL PART**

## Materials

2-(Dimethyl-amino)ethyl acrylate (DMAEA) was purchased from Sigma-Aldrich (Prague, Czech Republic) and distilled under vacuum prior to use. 4-Cyano-4-[(dodecylsulfanylthiocarbonyl)sulfanyl]pentanoic acid (TTC_12_-COOH) and 2-cyano-2-propyl-dodecyl trithiocarbonate (TTC_12_) (Sigma Aldrich, Prague, Czech Republic), and 2,2'-azobis(4-methoxy-2,4-dimethyl valeronitrile (V70, FUJIFILMS Wako Chemicals, U.S.A.) were used as received. Polymerization solvents *tert*-butanol and dimethylacetamide (DMA) (Sigma Aldrich, Prague, Czech Republic) were dried over molecular sieves.

1-ethyl-3-(3-dimethylaminopropyl)carbodiimide and propargylamine were products of TCI (Tokio, Japan). 4,4’-azobis(4-cyanopentanoic acid), 4-dimethylaminopyridine, acetic acid, ethanethiol, sodium hydride (NaH), carbon disulfide (CS_2_), potassium bisulfate (KHSO_4_), trifluoroacetic acid (TFA), sodium acetate, potassium dihydrogen phosphate, potassium hydroxide, phosphate monobasic monohydrate, potassium phosphate dibasic, [boric acid](https://www.sigmaaldrich.com/US/en/product/sigald/b0394?context=product), [sodium tetraborate](https://www.sigmaaldrich.com/US/en/product/aldrich/221732?context=product), sodium chloride were products of Sigma-Aldrich (Prague, Czech Republic) and were used as received. Acetone, dichloromethane, ethyl acetate, petroleum ether and magnesium sulfate **(**MgSO_4_) (Lachner s.r.o., Neratovice, Czech Republic) and sodium thiosulfate (Na_2_S_2_O_3_), diethyl ether and hexane (VWR, Stříbrná Skalice, Czech Republic) were used as received.

*Tert*-butanol-*d_10_*, acetone-*d_6_* (99.9% D), chloroform-*d* (99.8% D)*,* D_2_O (99.9% D) and trifluoroacetic acid-*d_1_* (≥ 99,5% D) were purchased from Eurisotop (Saint-Aubin, France).

## Synthesis of 2-Cyano-5-oxo-5-[(prop-2-yn-1-yl)amino]pentan-2-yl ethyl carbonotrithioate (TTC_2_-Pg)

TTC_2_-Pg was synthesized by modifying the procedure described by Johnson et al.^1^ Briefly, ethanethiol (5.63 mL, 76 mmol) was slowly added to a 60% oil suspension of NaH (3.15 g, 79 mmol) in diethyl ether (150 mL) under stirring at 0 °C. After 10 min, liquid CS_2_ (4.75 mL, 79 mmmol) was added, the formed *S*-ethyl trithiocarbonate was filtered (7.7 g, 47 mmol), resuspended in diethyl ether (100 mL) and allowed to react with I_2_ (5.96 g, 23.5 mmol) at room temperature until the solution became clear. The solid impurities were filtered off, the crude product was washed with aqueous Na_2_S_2_O_3_ (3 × 100 mL) and dried over a layer of MgSO_4_. After filtering off the desiccant and evaporating the solvent, solid bis(ethylsulfanylthiocarbonyl)disulfide (3.25 g, 12 mmol) was reacted with 4,4’-azobis(4-cyanopentanoic acid) (4.2 g, 15 mmol) in ethyl acetate (50 mL) under reflux at 80 °C for 18 h. The crude product was purified by column chromatography on silica gel using a mixture of ethyl acetate–hexane (1:1) as eluent to give 4.0 g (63%) g 4-cyano-4-(ethylsulfanylthiocarbonyl)sulfanylpentanoic acid as a yellow powder. ^1^H-NMR (CDCl_3_, δ): 1.36 t (3H, SCH_2_*CH_3_*), 1.88 s (3H, *CH_3_*) 2.30-2.65 m (4H, *CH_2_CH_2_*), 3.35 q (2H, S*CH_2_*CH_3_).

Next, 1-ethyl-3-(3-dimethylaminopropyl)carbodiimide (800.5 mg, 5.16 mmol) was dissolved in dichloromethane (8.0 mL) and added into a solution of propargylamine (267.4 μL, 4.18 mmol) and 4-cyano-4-ethyl-trithiopentanoic acid (1.0 g, 3.80 mmol) in dichloromethane (10 mL). A catalytic amount of 4-dimethylaminopyridine was added and the solution was stirred overnight at room temperature. The reaction mixture was then washed with 5% aqueous KHSO_4_ (1 × 25 mL) and H_2_O (1 × 25 mL) and dried over a layer of MgSO_4_. After filtering off the desiccant, the crude product was purified by column chromatography on silica gel using a gradient elution of ethyl acetate–hexane (1:3 🡪 1:1) to give 0.81 g (71%) 2-cyano-5-oxo-5-[(prop-2-yn-1-yl)amino]pentan-2-yl ethyl carbonotrithioate as a yellow powder. ^1^H-NMR (DMSO-*d6*, δ): 1.28 t (3H, SCH_2_*CH_3_*), 1.84 s (3H, *CH_3_*) 2.18-2.40 m (4H, *CH_2_CH_2_*), 3.11 t (1H, CH_2_C*CH*), 3.33-3.41 m (2H, *CH_2_*CCH), 3.86 q (2H, S*CH_2_*CH_3_), 8.46 t (1H, *NH*).

## Reversible addition–fragmentation chain transfer (RAFT) polymerization.

## DMAEA monomer

The example of polymerization conditions ([M]/[TTC_2_-Pg] = 260): the DMAEA monomer (1.5088 g, 10.54 mmol), *tert*-butanol (4.59 ml), and DMA (0.41 ml) were pipetted into a 10 ml tear-shaped flask equipped with a stirrer bar, the flask was sealed with a rubber septum, and the polymerization solution was degassed with a stream of argon for 15 minutes. Freshly prepared individual stock solutions of V70 initiator and CTA in DMA were mixed, and 0.1 ml of the final V70/CTA co-solution (CTA: 10.65 mg, 4.053 × 10^-2^ mmol; V70: 2.5 mg, 8.1 × 10^-3^ mmol) was added to the flask using Hamilton syringe under an argon atmosphere. The flask was placed into an oil bath and the polymerization was carried out under stirring at 40 °C for 1 to 14 hours. The flask was opened and immersed in an ice-cold bath to terminate the polymerization. The polymerization mixture was diluted with acetone and the product was precipitated in ice-cold petroleum ether using polyethylene beakers as the polymer stuck to glass surfaces. Precipitation in diethyl ether, hexane, or diethyl ether/petroleum ether mixture was not effective because it led to partial fractionation of the polymer, especially of the unprotonated PDMAEA, as detected by SEC analysis of the polymerization mixtures (at particular polymerization times) and corresponding isolated products. The product was isolated by centrifugation to form a yellowish viscous liquid, the supernatant was removed, and the obtained polymer was dried under vacuum overnight. Precipitation was carried out at least twice.

## DMAEA^+^/TFA^-^ monomer

The example of polymerization conditions ([M]/[TTC_2_-Pg] = 260): the monomer DMAEA (0.4715 g, 3.29 mmol), *tert*-butanol (1 ml) and DMA (0.22 ml) were added to 10 ml tear-shaped flasks equipped with a stirrer bar, the flask was sealed with a rubber septum, and the polymerization solution was degassed with a stream of argon for 15 minutes. Then, TFA (0.277 ml, 3.62 mmol, i.e., 1.1 molar excess with respect to the monomer) was added dropwise to the polymerization mixture using a Hamilton syringe immersed in the mixture under an argon atmosphere. The mixture was stirred for 10 minutes in an ice bath to protonate the amine groups of DMAEA side chains. Freshly prepared individual stock solutions of V70 initiator and CTA in DMA were mixed, and 0.1 ml of the final V70/CTA co-solution (CTA: 3.33 mg, 1.27 × 10^-2^ mmol; V70: 0.78 mg, 2.53 × 10^-3^ mmol) was added by a Hamilton syringe under an argon atmosphere. The reaction was carried out at 40°C under intensive stirring for 1 to 12 hours. For the product isolation, see Par. 2.1. The obtained products were hygroscopic, yellowish sticky tensile solids.

In all the experiments, the [CTA]/[I] ratio was 5, and the total content of DMA was 10 and 20% vol. for DMAEA and DMAEA^+^/TFA^-^, respectively. If required, aliquots of the polymerization mixture were collected under an argon atmosphere at specified time intervals and kept at -20 °C prior to ^1^H NMR and SEC analysis. The conversion was determined from the polymerization mixtures by ^1^H NMR spectroscopy in acetone-*d_6_*.

## *In situ* monitoring of RAFT polymerization by ^1^H-NMR

The polymerization mixtures of both DMAEA and DMAEA^+^/TFA^-^ monomers were prepared using deuterated *tert*-butylalcohol-*d_10_,* DMA and/or trifluoroacetic acid-*d_1_* as polymerization solvents in tear-shaped flasks using the same protocol as described for the standard polymerization (see, the Par. 2.1 and 2.2). The polymerization mixture was then transferred to an NMR tube filled with argon by the argon-purged syringe, the tube was sealed in an argon atmosphere and placed in the NMR instrument for the analysis of polymerization kinetics at 40 °C.

^1^H-NMR in-situ polymerization kinetics spectra were acquired with a Bruker Avance III 600 spectrometer operating at 600.2 MHz using “*zgdelay*” pulse program^2^ at 313 K. The width of the 90° pulse was 18 μs, the relaxation delay was 10 s, and the acquisition time was 2.18 s with 2 scans. Kinetics measurements were provided for 12 hours with time points every 10 minutes.

**Figs. S1 and S2** present high-resolution ^1^H-NMR spectra of DMAEA and DMAEA^+^/TFA^-^ polymerization solutions in *tert*-butanol-*d*_6_ and *tert*-butanol-*d*_6_/TFA-*d_1_*, respectively, which were recorded at three-time points of the polymerization, including peak assignments of proton types used to calculate the conversions. The conversion of both monomers was calculated from the ratio of the integral intensity of CH_2_ double bond signals of monomers (**a_1_’)** and CH_2_ groups adjacent to the oxygen in monomer (**c’**) and polymer (**c**). Because the signal position of CH_2_ groups of monomers and polymers (**c’**, **c**) is almost the same, the total integral intensity of these signals (i.e., **c’ +** **c**) was set as “2”. Afterward, the monomer conversion was calculated by using the equation:

Conv.(%)=100 × (1-I_a1’_)

The integrated intensities were determined with TopSpin 4.0.5 software.

## Determination of monomer conversion by ^1^H-NMR analysis in acetone-*d_6_*

100 μl aliquots of polymerization mixtures were dissolved in 500 μl of acetone-*d_6_* and provided for ^1^H NMR analysis using Bruker Avance Neo 400 spectrometer operating at 400.1 MHz. The width of the 90° pulse was 16.5 μs, the relaxation delay was 10 s, and the acquisition time was 3.41 s with 32 scans.

The monomer conversion was calculated from the ratio of the integrated signal of the CH_2_ protons of the vinyl group in monomers at 6.38 (DMAEA) and 6.47 (DMAEA^+^/TFA^-^) ppm to the integrated signal at 4.08 - 4.32 (DMAEA) and 4.66 - 4.45 (DMAEA^+^/TFA^-^) ppm corresponding to the methylene protons adjacent to the oxygen in both the monomer and polymer (constant throughout the polymerization). The integrated intensities were determined with TopSpin 4.0.5 software with an accuracy of ±1%. The monomer conversion was calculated as described in Par. 2.3.

Representative ^1^H NMR spectra of PDMAEA and PDMAEA^+^/TFA^-^ polymerization mixtures in acetone-*d*_6_ are presented in **Figs. S3** and **4**, respectively.

## Size exclusion chromatography analysis

Unprotonated PDMAEA polymers: Weight-average molecular weight (*M*_w_), number-average molecular weight (*M*_n_), and dispersity (*Ð*) were measured using size exclusion chromatography (SEC) on an HPLC Shimadzu system series 40 equipped with an SPDM40 photodiode array detector (Shimadzu, Japan) with two different detectors, i.e., differential refractometer (Optilab), multiangle light-scattering (DAWN-8) detector (both from Wyatt Technology Co., USA), a DGU-403 degasser, an LC-40D pump, and columns PSS Gram combination medium in series (1x Gram precolumn + 1x Gram column 30Å + 2x Gram column 1000Å (PSS polymer standard Services GmbH, Germany). The system was controlled by a CBM-40 unit. The samples were injected with a SIL-40C autosampler unit and measured in a DMF/ 0.01 M LiBr mobile phase at a flow rate of 1 ml/min. A method based on a known total injected mass assuming 100% mass recovery was used to estimate the d*n*/d*c* value (0.045 ml/g) to calculate the molecular weights of the polymers from the light scattering data.

Protonated PDMAEA^+^/TFA^-^ polymers: Weight-average molecular weight (*M*_w_), number-average molecular weight (*M*_n_), and dispersities (*Ð*) were determined by SEC on an HPLC system (Shimadzu, Japan) equipped with an internal UV-VIS diode array detector (SPD-M20A), external differential refractometer (Optilab T-rEX, Wyatt Technology, USA) and multiangle light scattering detector (DAWN HELEOS II, both Wyatt Technology, USA). Gram precolumn + TSKgel G3000PWXL-CP (7.8 x 300 mm, Chromservis s.r.o., Prague, Czech Republic) and a mobile phase of 0.1 M NaNO_3_/TFA, pH 2.5, at a flow rate of 1 ml/min were used to analyze the charged samples. A method based on a known total injected mass assuming 100% mass recovery was used to estimate the d*n*/d*c* value (0.127 ml/g) to calculate the molecular weights of the polymers from the light scattering data.

## Hydrolysis studies

The 3.5 mg of PDMAEA and/or PDMAEA^+^/TFA^-^ were dissolved in 0.7 ml of 100 mM acetate (pH 5, 6), citrate-phosphate (pH 5), phosphate (pH 5 and 7.4) and borate- (pH 9) buffered D_2_O solution, giving a final 0.5 % wt polymer solution. The samples were transferred to NMR cuvettes, carefully sealed to avoid evaporation, and placed in a thermostat at 37 °C for three weeks. At predetermined time intervals, ^1^H NMR spectra were recorded with Bruker Avance Neo 400 spectrometer operating at 400.1 MHz. The width of the 90° pulse was 16.5 μs, the relaxation delay 10 s, and acquisition time 3.41 s with 64 scans.

Hydrolytic degradation during the first three days at pH 7.4 and 9 was followed by the *in situ* ^1^H-NMR monitoring by Bruker Avance III 600 spectrometer operating at 600.2 MHz at 37 °C. The width of the 90° pulse was 18 μs, the relaxation delay 10 s, and acquisition time 2.18 s with 64 scans.

The rate of hydrolysis was calculated from the ratio of the integrated signals at 3.92 ppm corresponding to methylene groups adjacent to the oxygen of dimethylaminoethanol (DMAE, the degradation product) and a broad signal with a maximum at 4.4 (PDMAEA) and/or 4.45 (PDMAEA^+^/TFA^-^) ppm corresponding to the methylene protons adjacent to the oxygen of polymers. The representative ^1^H NMR spectra for PDMAEA^+^/TFA^-^ in a phosphate-buffered D_2_O solution (pH 7.4) after 10 minutes, 1, 7 and 21 days are shown in **Fig. S8.**

The integrated intensities were determined with TopSpin 4.0.5 software with an accuracy of ± 1%.

Determination of pH values: the determined pD values of the polymer solutions in buffered D_2_O after three weeks of incubation were adjusted by adding 0.45 from the recorded pH value.^3^

**References:**

(1) Johnson, R. N.; Burke, R. S.; Convertine, A. J.; Hoffman, A. S.; Stayton, P. S.; Pun, S. H. Synthesis of Statistical Copolymers Containing Multiple Functional Peptides for Nucleic Acid Delivery. *Biomacromolecules* **2010**, *11* (11), 3007-3013. DOI: 10.1021/bm100806h.

(2) Abbrent, S.; Mahun, A.; Smrčková, M. D.; Kobera, L.; Konefał, R.; Černoch, P.; Dušek, K.; Brus, J. Copolymer chain formation of 2-oxazolines by in situ 1H-NMR spectroscopy: dependence of sequential composition on substituent structure and monomer ratios. *RSC Adv.* **2021**, *11* (18), 10468-10478, DOI: 10.1039/D1RA01509E.

(3) Krezel, A.; Bal, W. A formula for correlating p*K*_a_ values determined in D_2_O and H_2_O. *J. Inorg. Biochem.* **2004**, 98, 161−166.

# **RESULTS**


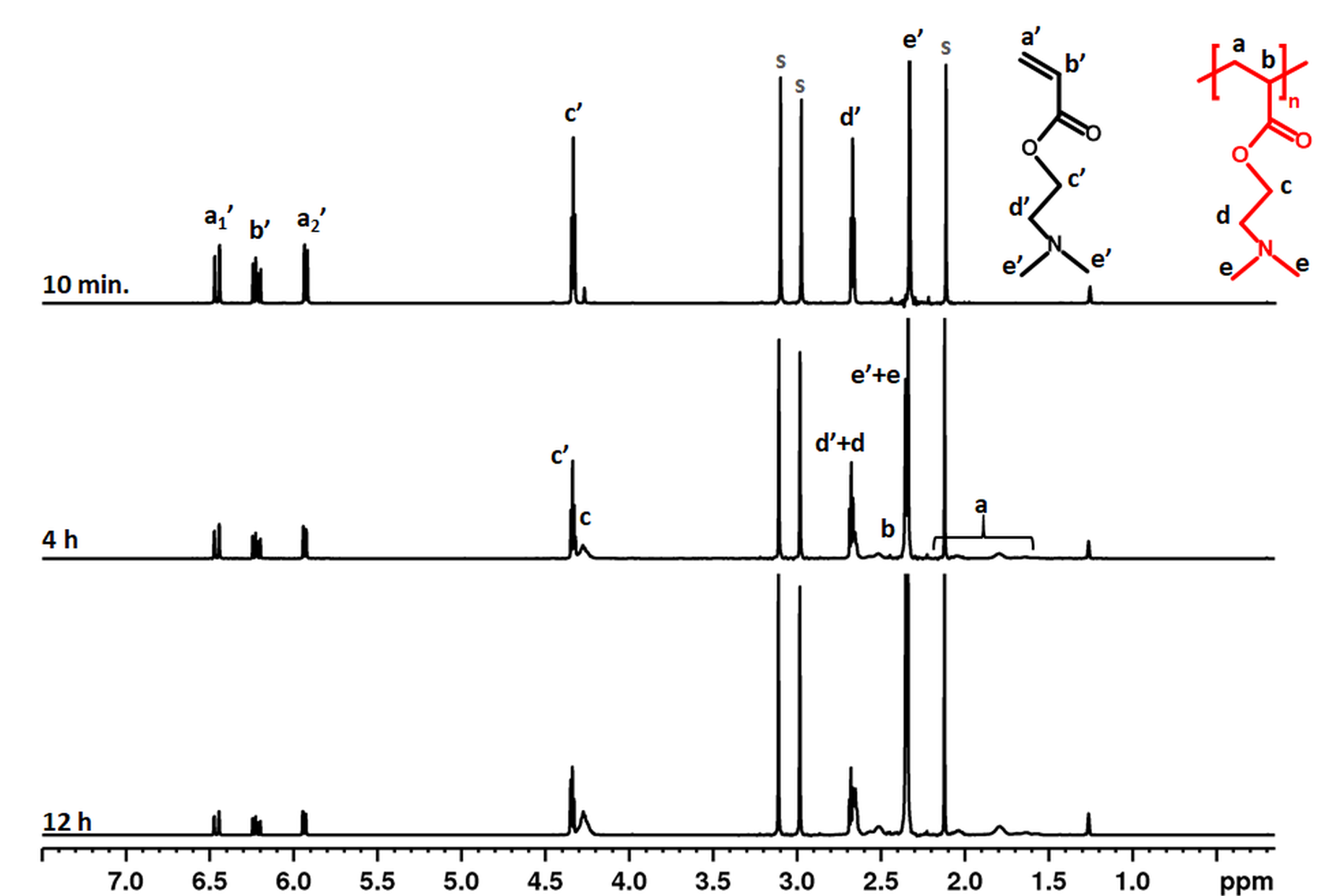


**Fig. S1** ^1^H-NMR spectra of the PDMAEA polymerization solution in *tert-*butanol-*d*_10_ recorded at 313 K after 10 minutes, 4 and 12 hours of polymerization under the same instrumental conditions. “s” – solvent peak


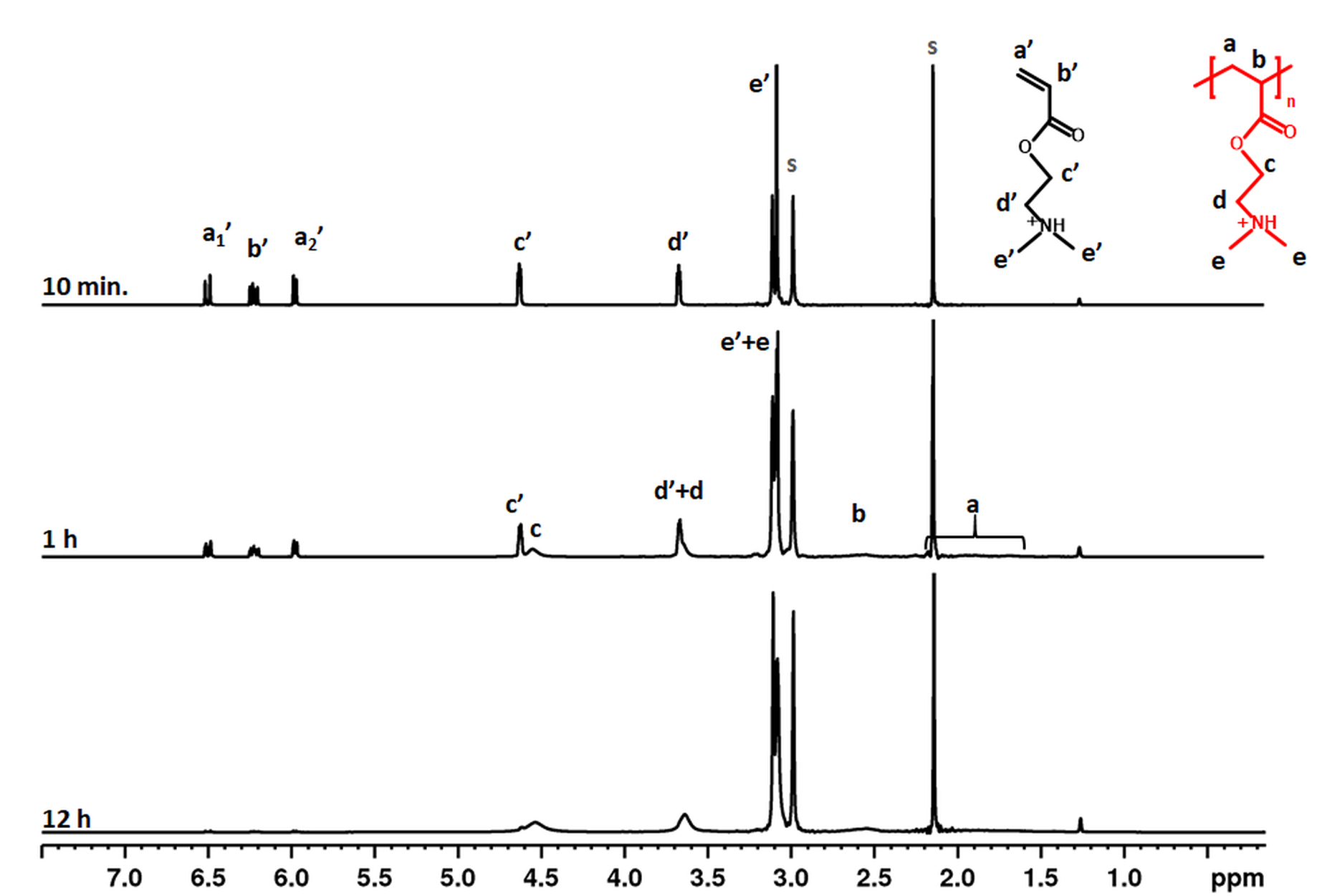


**Fig. S2** ^1^H-NMR spectra of the PDMAEA^+^/TFA^-^ polymerization solution in a *tert-*butanol-*d*_10_/TFA-*d*_1_ mixture recorded at 313 K after 10 minutes, 1 and 12 hours of polymerization under the same instrumental conditions. “s” – solvent peak

**
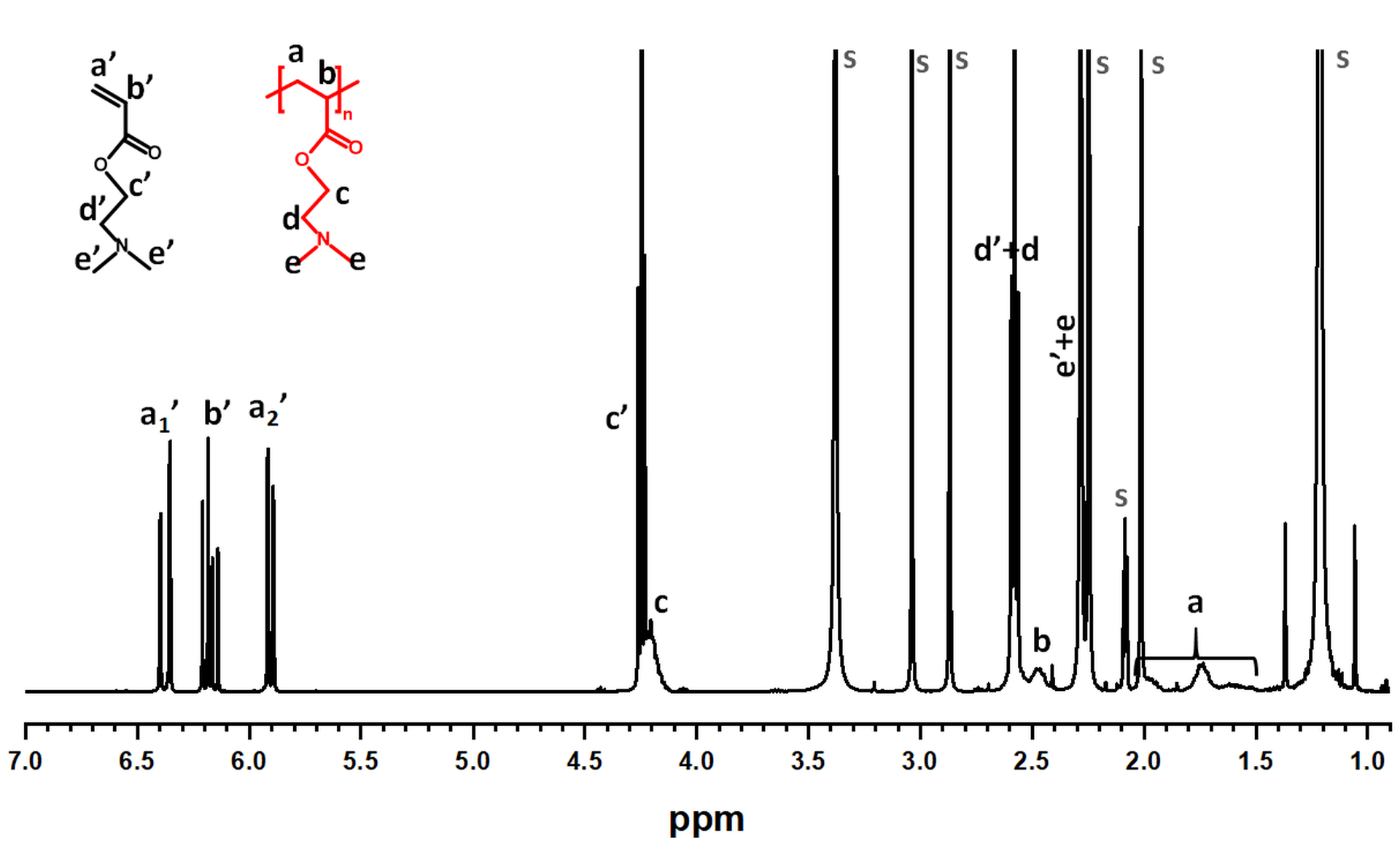
**

**Fig. S3** ^1^H-NMR spectra of the PDMAEA polymerization solution in acetone-*d*_6_ recorded at 299 K after 6 h of polymerization. “s” – solvent peak


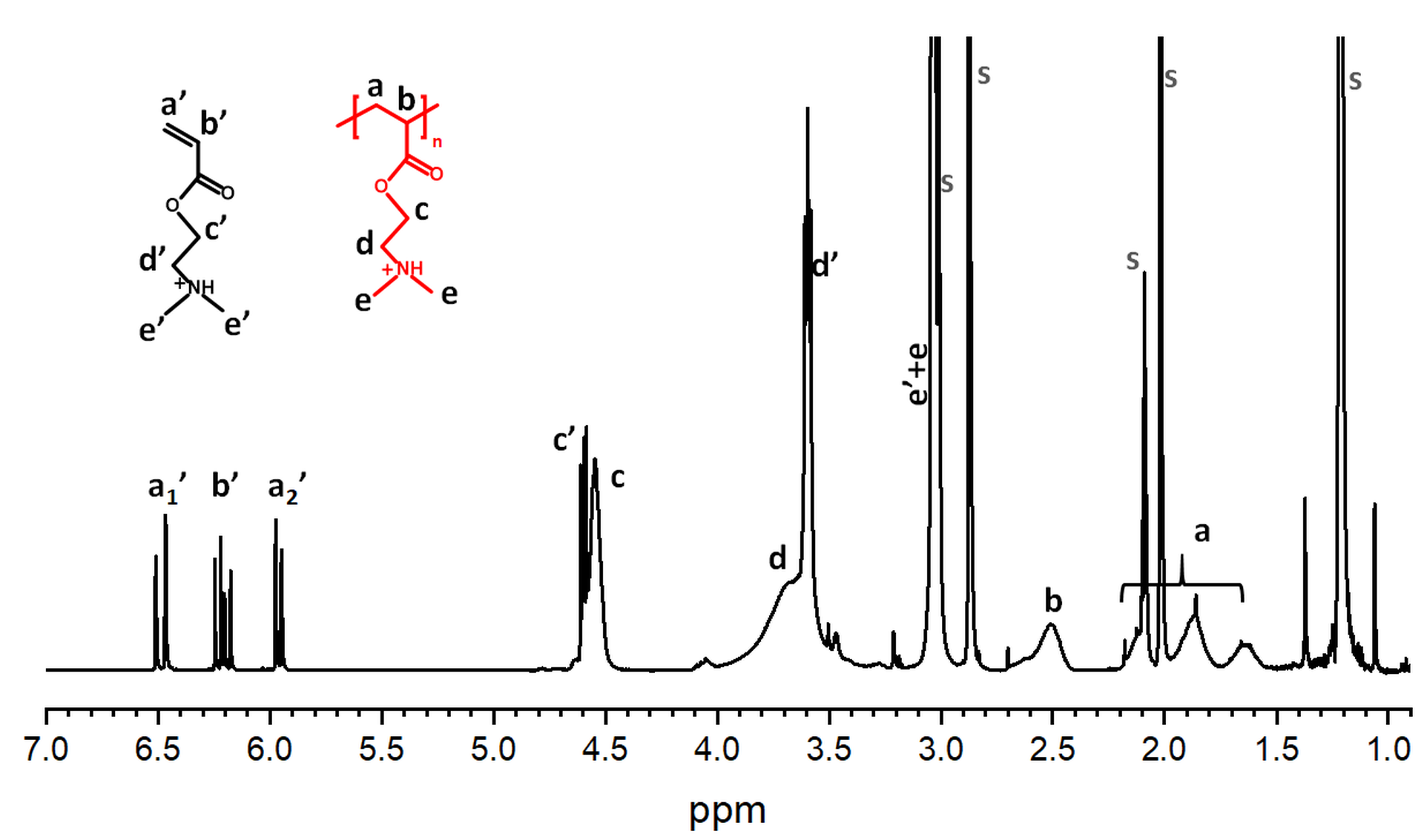


**Fig. S4** ^1^H-NMR spectra of the PDMAEA^+^/TFA^-^ polymerization solution in acetone-*d*_6_ recorded at 299 K after 50 minutes of polymerization. “s” – solvent peak


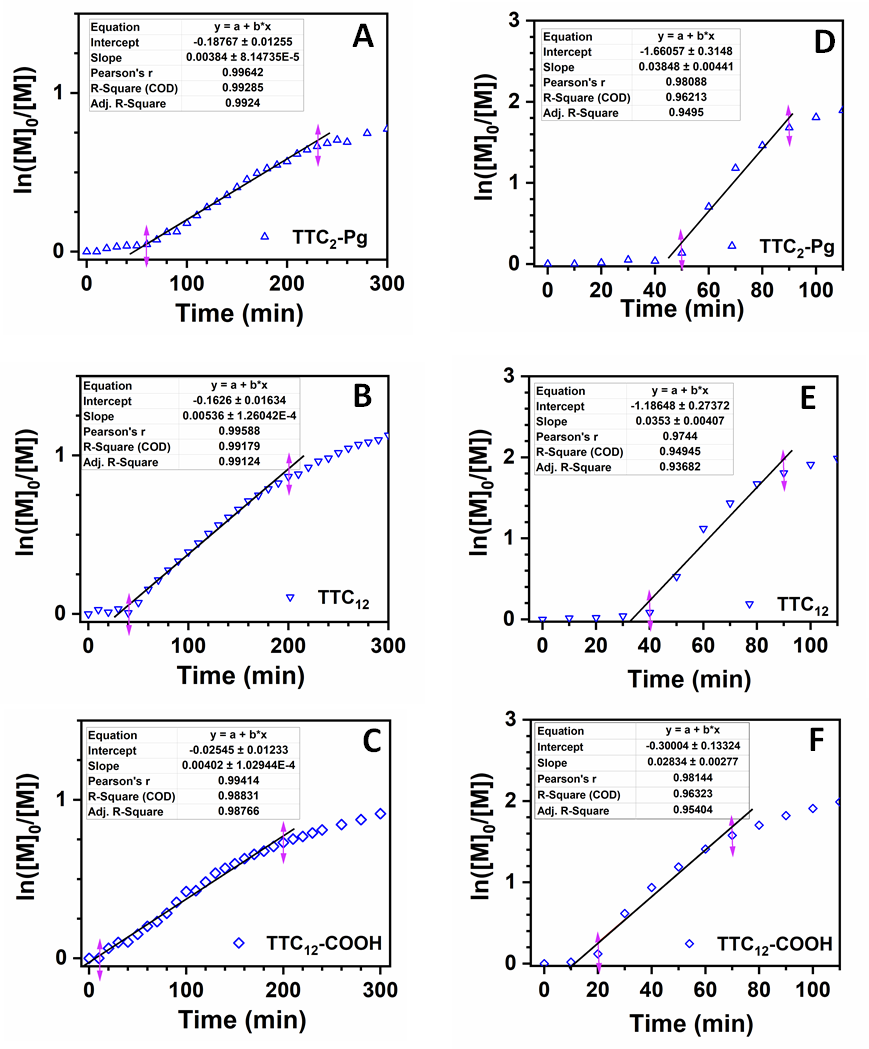


**Fig. S5** The linear fitting of the kinetic plots of ln([M_0_]/[M]) versus time for PDMAEA (A-C) and PDMAEA^+^/TFA^-^ (D-F) polymerized in the presence of TTC_2_-Pg (A, D), TTC_12_ (B, E) and TTC_12_-COOH (C, F) as the CTA.

## **Table S1** Effect of [M]/[TTC_2_-Pg] molar ratio on the molecular weight of PDMAEA and PDMAEA^-^/TFA^+^.

| Polymer | [M]/[TTC_2_-Pg] | *M*_n-theor_ ^a^ | *M*_w_ ^b^ | *M*_n_ ^b^ | *Ð* ^b^ | Conversion ^c^ |
| --- | --- | --- | --- | --- | --- | --- |
|  | (mol/mol) | (g/mol) | (g/mol) | (g/mol) |  | (%) |
| PDMAEA ^d^ | 50 | 7 200 | 9 200 | 8 400 | 1.12 | 89 |
|  | 100 | 14 300 | 18 400 | 16 800 | 1.13 | 86 |
|  | 260 | 37 200 | 32 800 | 27700 | 1.19 | 69 |
|  | 300 | 42 900 | 33 500 | 28 500 | 1.21 | 70 |
|  | 400 | 57 300 | 37 600 | 31 500 | 1.23 | 66 |
|  | 500 | 71 600 | 40 800 | 32 500 | 1.25 | 66 |
|  | 600 | 85 900 | 45 100 | 36 400 | 1.29 | 65 |
| PDMAEA^+^/TFA^-d^ | 50 | 12 900 | 11 900 | 10 600 | 1.03 | 90 |
|  | 100 | 25 700 | 24 500 | 22 400 | 1.09 | 90 |
|  | 260 | 66 900 | 66400 | 62 000 | 1.07 | 94 |
|  | 300 | 77 200 | 77 300 | 71 600 | 1.08 | 98 |
|  | 400 | 102 900 | 101 200 | 90 400 | 1.12 | 97 |

M - monomer, TTC_2_-Pg - CTA agent, ^a^… Theoretical molecular weight based on a [M]/[TTC_2_-Pg] molar ratio in the polymerization mixture calculated for 100% conversion; ^b^… Weight (*M*_w_) and number (*M*_n_) average molecular weight, and dispersity (*Ð*) of the products determined by Size exclusion chromatography (see the **SI** for details about measurement conditions); ^c^… calculated from ^1^H NMR analysis in acetone-*d_6_*. (see **Figs. S3** and **S4** for illustrative ^1^H NMR spectra); ^d^…polymerization time: PDMAEA - 9 h, and PDMAEA^+^/TFA^-^ - 2 h; [TTC_2_-Pg]/[V70] = 5 mol/mol, [M] = 2 M in *tert*-butanol/DMA 10% vol (DMAEA) and *tert*-butanol/DMA 20% vol (DMAEA^+^/TFA^-^) solvent mixtures, 40 °C;


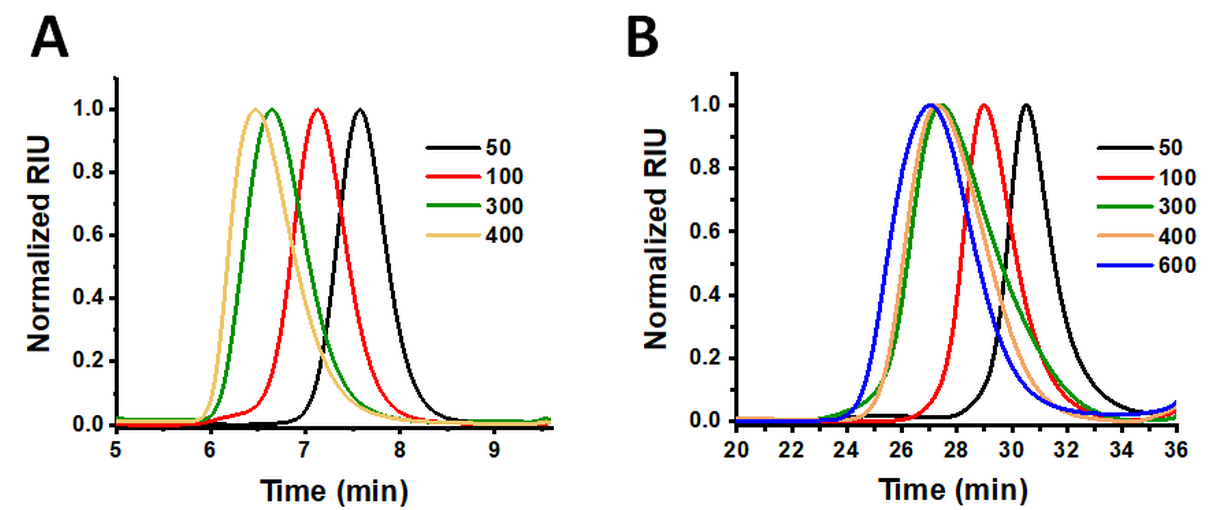


**Fig. S6** Normalized SEC chromatograms of protonated PDMAEA^+^/TFA^-^ (A) and unprotonated PDMAEA (B) polymers prepared at a [M]/[TTC_2_-Pg] ratio of 50 (black line), 100 (red line), 300 (green line), 400 (yellow line) and 600 (blue line). Common polymerization conditions: [M] = 2 M, [TTC_2_-Pg]/[V70] = 5 mol/mol, 40 °C; Specific conditions for PDMAEA^+^/TFA^-^: in *tert*-butanol/DMA 20% vol. and 2 hours, SEC analysis using a 0.1M NaNO_3_/TFA mobile phase (pH 2.5); Specific conditions for PDMAEA: in *tert*-butanol/DMA 10% vol. and 12 hours, SEC analysis using a DMF/0.01 M LiBr mobile phase.


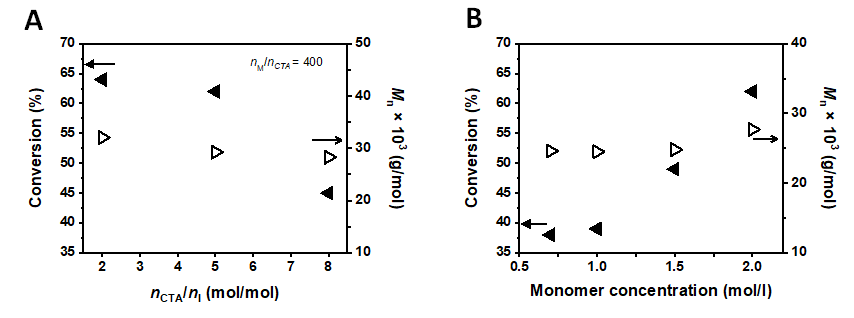


**Fig. S7** Polymerization of the unprotonated PDMAEA monomer. Dependence of monomer conversion () and *M*_n_ () of products on the [TTC_2_-Pg]/[V70] ratio (A) and the DMAEA concentration in polymerization mixture (B). Polymerization conditions: in *tert*-butanol/DMA 10% vol, 40 °C, 12 hours; for graph A: [DMAEA] = 2 M; [DMAEA]/[TTC_2_-Pg] = 400; for graph B: [TTC_2_‑Pg]/[V70] = 5 mol/mol; SEC analysis using a DMF/LiBr mobile phase.


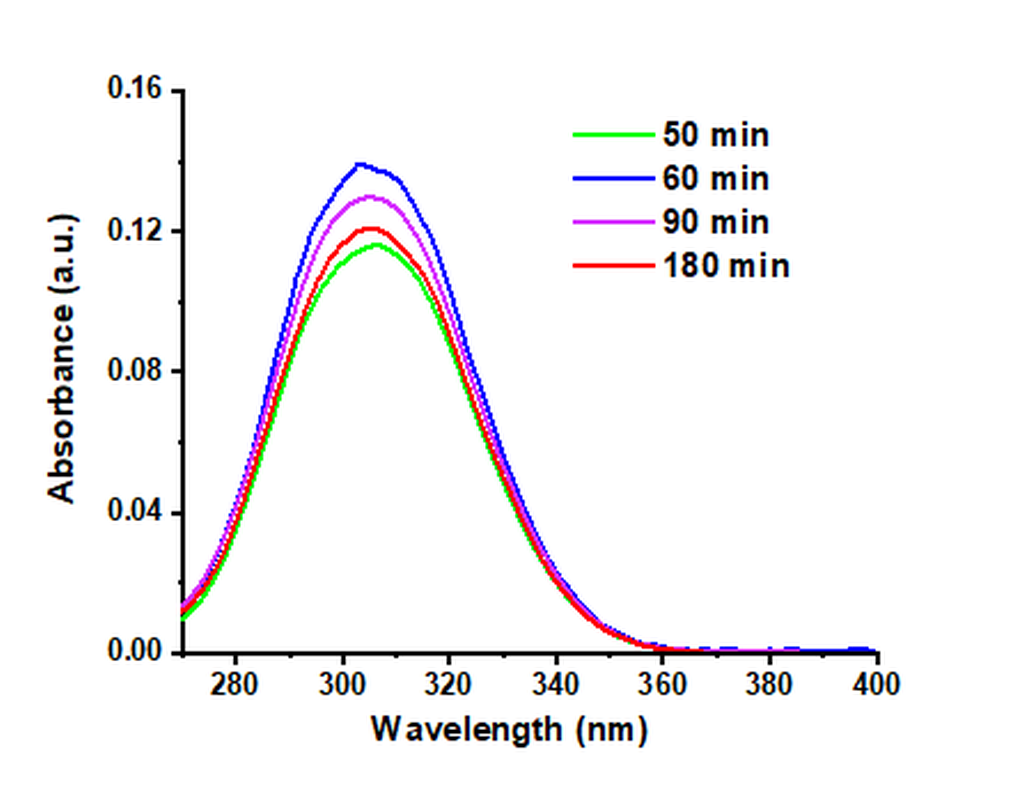


## **Fig. S8** Polymerization of the protonated DMAEA^+^/TFA^-^. Absorption band intensity at a wavelength of 307 nm (corresponding to the polymer TTC-containing CTA end groups) at various polymerization times.


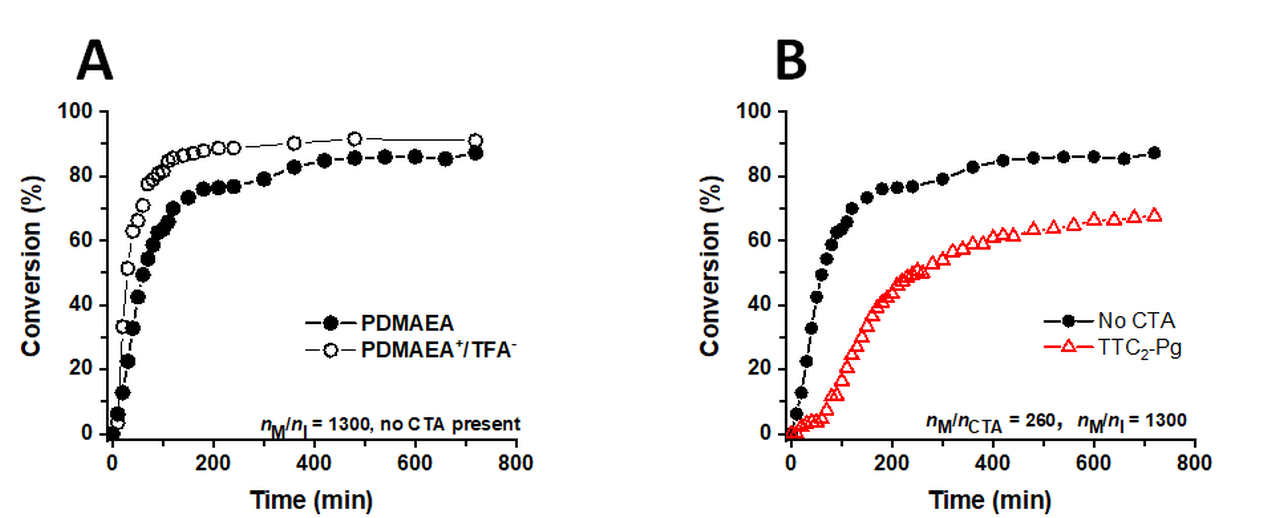


**Fig. S9** Free radical polymerization of DMAEA and DMAEA^+^/TFA^-^ monomers in *tert*-butanol/DMA with V70 initiator: (A) Dependence of the conversion on polymerization time for free radical polymerization of DMAEA () and DMAEA^+^/TFA^-^ (); (B) Dependence of the conversion on polymerization time for free radical () and RAFT () polymerization of DMAEA with TTC_2_-Pg as CTA. [M] = 2 M in *tert*-butanol-*d*_10_/DMA 10% vol. (DMAEA) and *tert*-butanol‑*d*_10_/DMA 20% vol. (DMAEA^+^/TFA^-^) solvent mixtures, 40 °C. ^1^H NMR analysis in a a *tert-*butanol-*d*_10_ (PDMAEA) and tert-butanol-*d*_10_/TFA-*d*_1_ mixture (PDMAEA^+^/TFA^-^)


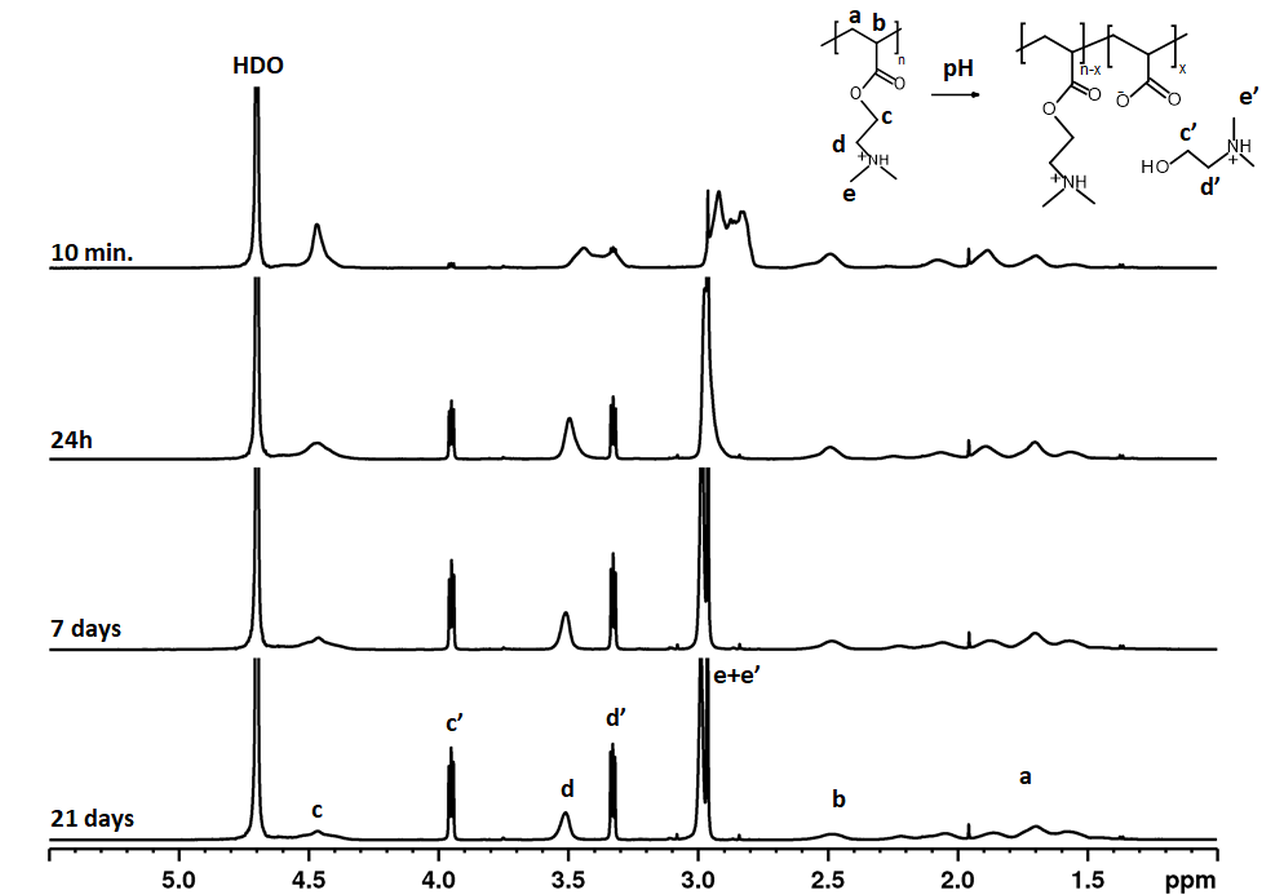


**Fig. S10** ^1^H-NMR spectra of PDMAEA^+^/TFA^-^ in a phosphate-buffered D_2_O solution (pH 7.4) after 10 minutes, 1, 7, and 21 days of incubation at 37 °C.

| Buffer, initial pH | pH of the polymer solution after 3-week hydrolysis | |
| --- | --- | --- |
|  | PDMAEA | PDMAEA^+^/TFA^-^ |
| Borate buffer, pH 9 | 9.2 | 9.0 |
| Phosphate buffer, pH 7.4 | 7.9 | 7.5 |
| Acetate buffer, pH 6 | 9.3 | 6.0 |
| Acetate buffer, pH 5 | 9.3 | 5.5 |
| Acetate buffer, pH 5, (1 M) | 7.8 | x |
| Phosphate buffer, pH 5 | 6.4 | x |
| Citrate-phosphate buffer, pH 5 | 6.7 | x |

## **Table S2** The pH values of PDMAEA and PDMAEA^+^/TFA^-^ solutions in 0.1 M buffers of various pH at 37 °C after 3-week hydrolytic degradation.


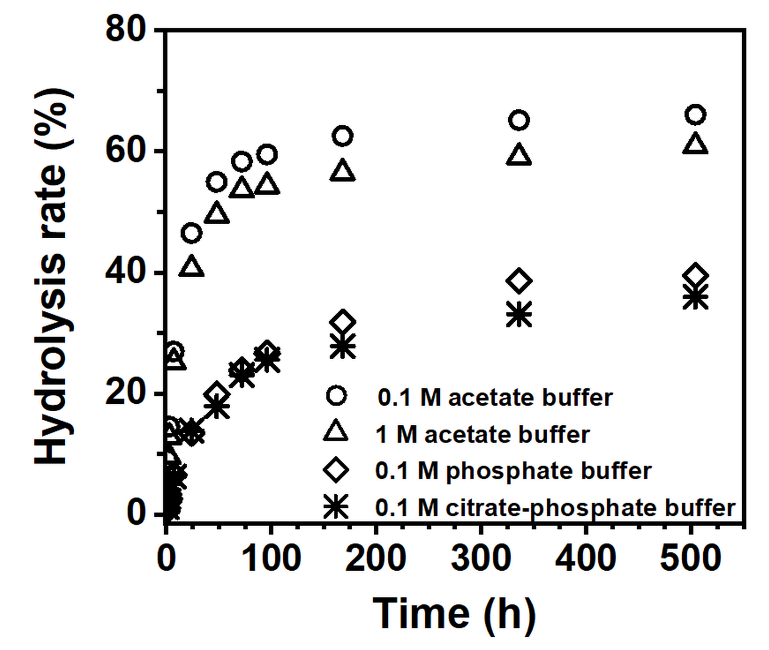


**Fig. S11** Hydrolysis of a 0.5% wt. PDMAEA solution at pH 5 using 0.1 M acetate, phosphate and citrate-phosphate-buffered D_2_O, and 1 M acetate-buffered D_2_O at 37 °C. (^1^H-NMR analysis).
